# Comparative Analysis of Dental Age Estimation: A Systematic Review and Meta-analysis Assessing Gender-Specific Accuracy of the Demirjian and Nolla Methods Across Different Age Groups

**DOI:** 10.7759/cureus.75031

**Published:** 2024-12-03

**Authors:** Abdulkreem Al-Juhani, Abdulaziz Binshalhoub, Saleh Showail, Mofareh Alraythi, Abdulrahman Alzahrani, Norah F Almutiri, Raghad F Alrasheed, Mohammed J Alzahrani

**Affiliations:** 1 Surgery, King Abdulaziz University Faculty of Medicine, Jeddah, SAU; 2 Forensic Medicine, Forensic Medicine Center, Riyadh, SAU; 3 Forensic Medicine, Forensic Medicine Services Administration, Riyadh, SAU; 4 Medicine, Faculty of Medicine, Jazan University, Jazan, SAU; 5 Medicine, Taif University, Taif, SAU; 6 General Medicine, Unaizah College of Medicine, Qassim University, Qassim, SAU; 7 Medicine, Alfaisal University College of Medicine, Riyadh, SAU; 8 Medicine, King Abdulaziz University Faculty of Medicine, Jeddah, SAU

**Keywords:** chronological age, demirjian, dental age, meta-analysis, nolla

## Abstract

Chronological age (CA) estimation is essential in medicine, forensics, and law. Teeth are often used for this due to their reliability. The Demirjian and the Nolla methods are used to estimate dental age (DA). Both methods have strengths and weaknesses; the Demirjian method usually overestimates age, whereas the Nolla method underestimates it. Their accuracy varies among different populations. Our objective is to compare the accuracy of these methods across various age groups and to probe the effect of gender. We searched PubMed, Scopus, the Excerpta Medica database (EMBASE), the Cochrane Library, and the Web of Science for relevant articles until March 2024. We then screened for comparative studies using the Demirjian and the Nolla methods. We used the RevMan 5.4 software package (The Cochrane Collaboration, London, UK) to compare the accuracy of both methods in estimating chronological age in both genders across age groups ranging from five to 16 years. All data were pooled using a random effects model, and relevant forest plots were generated. The accuracy was calculated based on the pooled mean difference between the chronological age and that estimated by each method. Our literature search identified 25 articles for inclusion in the review. The Demirjian method overestimated the age in males by 0.71 years in the six to 6.99 age group and an average overestimation of 0.5 years across all age groups. In females, the overestimation was 0.82 years in the 11-11.99 age group, but the average overestimation was 0.5 years. Overall, the Nolla method underestimated the age of males by 0.28 years and females by 0.25 years. Estimations from both methods were 0.7 years apart on average. In conclusion, while the Demirjian and Nolla methods have unique advantages, using them together can provide a more robust and reliable age estimation. Forensic practitioners can determine the best approach by considering population-specific accuracy, age group and gender, and the case context. Combining both techniques offers cross-verification, comprehensive assessment, bias mitigation, and enhanced reliability.

## Introduction and background

Chronological age (CA) estimation involves predicting an individual's age based on the time elapsed since birth. It is crucial in various fields, such as medicine, forensic anthropology, and jurisdiction. Predicting developmental stage, growth, and overall health in clinical settings is vital, particularly in pediatric care and orthodontics [1-3]. This aids healthcare professionals in devising age-appropriate treatment plans and understanding age-related norms and milestones [2, 3]. In forensics, CA estimation assists in recognizing unidentified individuals or victims by analyzing their skeletal remains and dental structures [4]. Additionally, CA estimation is vital for administrative and legal purposes, facilitating the determination of legal adulthood in undocumented individuals and ensuring fair outcomes in judicial and administrative decisions [2, 5].

Estimation of CA is possible through various methods, and body parts differ across disciplines. The body parts employed for age estimation include teeth, skeletal structures, femoral notch width, medial tibial depth, and tibial spine height [6, 7]. Furthermore, two methods exist for CA estimation depending on the discipline involved. Dental age (DA) estimation is preferred in forensic dentistry and orthodontics, whereas bone age assessment is extensively used in pediatric medicine [8-10]. Dental age estimation offers distinct advantages in reliability and objectivity. Teeth have inherent durability and resistance to taphonomic processes and post-mortem changes. Unlike other body parts, teeth maintain structural integrity over time, making them reliable age indicators [7, 11]. Dental age estimation methods, like the Demirjian and Nolla methods, are commonly used to estimate CA in children by evaluating tooth development and eruption patterns [2, 12, 13].

Different challenges arise during DA estimation. These include variability in tooth development, potential for age underestimation or overestimation, presence of dental anomalies, gender differences, and the significant impact of ethnicity on dental maturity [14-19]. Addressing these challenges requires the development of more nuanced and adaptable age estimation models that consider individual variability, dental anomalies, and ethnic-specific differences to improve the accuracy and reliability of DA assessment across various populations [20, 21].

The Demirjian method and the Nolla method are two techniques used in DA estimation, each with its unique use cases as well as downsides. The Demirjian method, developed in 1973, evaluates the stages of development of seven left mandibular teeth to estimate an individual's age by assigning maturity scores based on specific developmental stages [22, 23]. This method is recommended for its objectivity, ease of use, systematic approach, and high accuracy across diverse populations [22-24]. Additionally, research has demonstrated that methods such as Demirjian's method tend to overestimate DA in both boys and girls [25]. Compared to the Nolla method, the Demirjian method performs more in specific populations, such as North Indian children [26, 27].

On the other hand, the Nolla method, developed in 1960, focuses on analyzing the calcification stages of individual teeth, assigning each tooth to a specific stage ranging from 0 to 10 based on its developmental status [28]. The Nolla method provides a systematic approach to assessing dental maturity and is widely utilized in teaching, clinical practice, and research in forensic sciences and orthodontics [11, 29-31]. One noteworthy pitfall of the Nolla method is that it sometimes underestimates age, as observed in certain studies [32, 33]. Despite potential limitations in precision, the Nolla method remains a valuable tool for DA estimation, providing insights into tooth calcification stages and aiding in age assessment in various populations [34].

These two methods have demonstrated varying levels of accuracy in predicting CA, with some showing discrepancies in specific populations [3, 35]. When looking up the current state of the literature, we came across a previous study comparing the Demirjian and Willems methods [12]. Another study investigated multiple methods of DA estimation, including the Nolla and Demirjian methods; however, the population was confined to Brazilians [36]. In this systematic review, we aim to delineate the accuracy of these methods separately and then compare their performance across different age groups.

## Review

Methods

This systematic review was conducted following the Preferred Reporting Items for Systematic Reviews and Meta-Analyses (PRISMA) and as outlined in the Cochrane Handbook for Systematic Reviews of Interventions [37, 38].

The inclusion criteria consisted of cross-sectional studies that explore both methods and have their full text available. Additionally, the study should encompass participants aged between 0 and 16 years. To ensure the accuracy of the study, several exclusion criteria were applied: studies with incomplete or missing data, inconsistent comparison groups, and those not conducted in English.

Information Sources and Search Strategy

Record retrieval went through three stages. We first searched PubMed, Scopus, and the Excerpta Medica database (EMBASE) for relevant articles using generic terms. Once this was obtained, we formulated a search strategy utilizing all relevant terms from the first step. The second stage involved searching PubMed, Scopus, EMBASE, the Cochrane Library, and Web of Science using the following terms: ((Tooth OR Teeth OR Dent* OR Odont*) AND ("Age Estimation" OR "Age Determination*" OR "Age Calculation" OR "Age Prediction") AND (Demirjian* OR Nolla*)). The search was conducted on March 7, 2024. Finally, we conducted a manual search using the references and citations of the records retrieved from the previous stage to look for other relevant studies.

Selection Process

Two independent reviewers screened titles and abstracts from all available records; a third reviewed conflicts. Again, the same reviewers who conducted the initial screening checked the eligibility of articles included from the previous step in full text, and disputes were resolved by discussion.

Data Extraction

Two independent reviewers examined all articles retrieved from full-text screening. The data were then stored in a Microsoft Excel spreadsheet (Microsoft Corp., Redmond, WA). A third reviewer checked concordance and resolved conflicts as necessary.

Outcomes and Effect Measures

Baseline and summary information, including author names, study settings, participant numbers, and gender distribution, was extracted from all eligible articles.

We assessed three outcomes: 1. The discrepancy between the Demirjian and Nolla methods in males; 2. The disparity between the Demirjian and Nolla methods in females; 3. The mean age difference between the Demirjian method and the Nolla method.

The previous outcomes were compared according to gender and age groups across different populations. All outcomes were expressed as mean differences.

Risk of Bias

Two independent reviewers, blinded to author names and affiliations, evaluated the methodological quality of all eligible articles using the Strengthening the Reporting of Observational Studies (STROBE) checklist [39]. This is a 40-item checklist used to assess observational studies. Studies scoring 33 or higher were categorized as having high methodological quality. Scores ranging from 22 to 32 were deemed moderate quality, while scores below 21 indicated low quality. Conflicts were later resolved through discussion.

Statistical Analysis

The analysis was undertaken using the RevMan 5.4 software package (The Cochrane Collaboration, London, UK). Mean differences for different age groups were pooled separately. In addition, we explored the data for both genders individually and collectively. We pooled effect sizes for each outcome as a mean difference by employing a random-effects model and inverse-variance method for weighting. A p-value less than 0.05 was considered significant. The last outcome, which involves the comparison of the Demirjian and the Nolla methods, was investigated using a student t-test to identify potential differences in the mean produced by each method. Heterogeneity was estimated using I­2 and the p-value of tau2; a value of I­2 exceeding 50% together with a p-value of tau2 less than 0.05 represented a significant heterogeneity. Forest plots and 95% confidence intervals were generated for the outcomes across different subgroups.

Results

Study Selection

**Figure 1:**
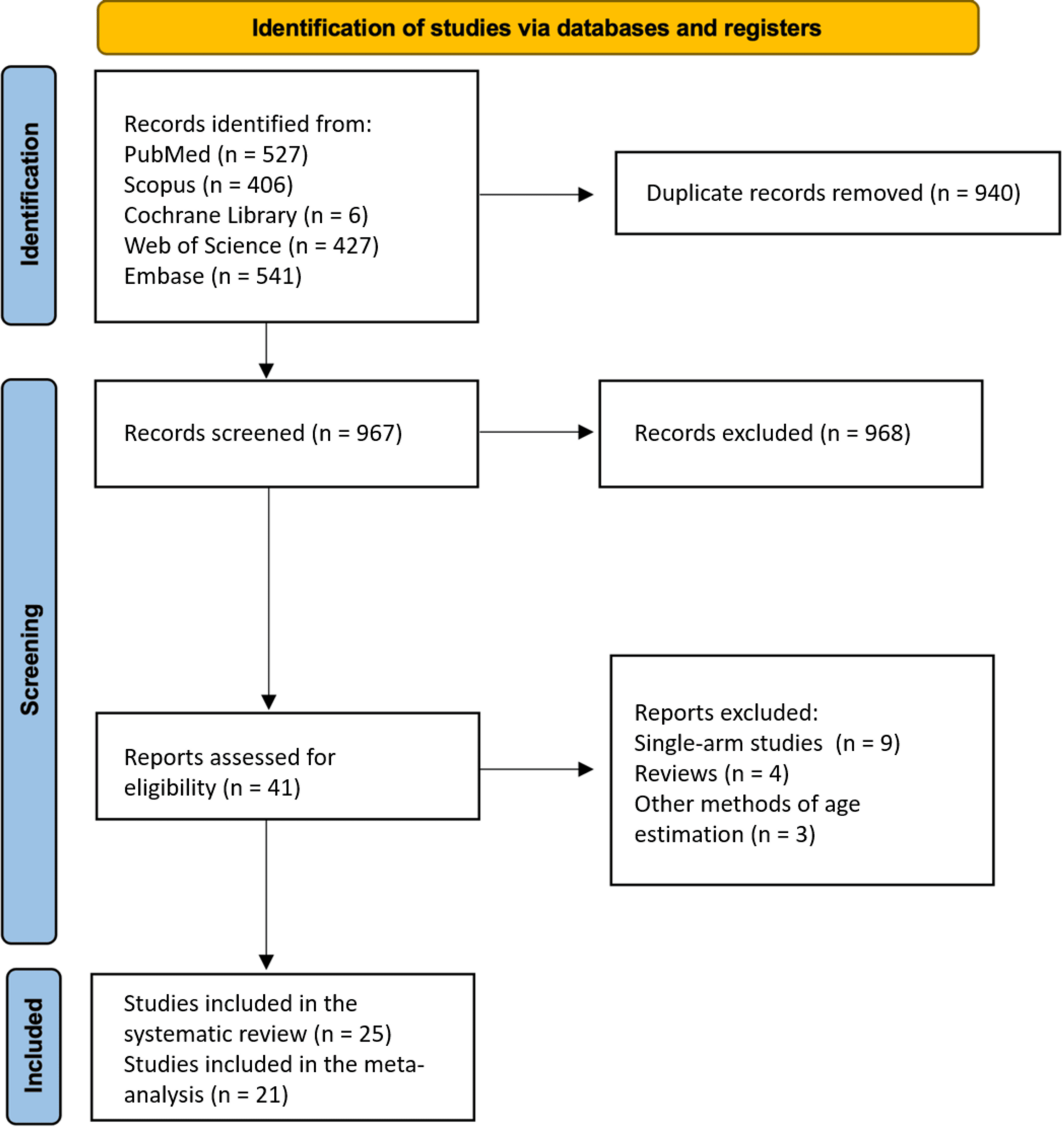
A PRISMA flowchart outlining the study selection process The PRISMA 2020 flow diagram for new systematic reviews included searches of databases and registers only. PRISMA: Preferred Reporting Items for Systematic Reviews and Meta-Analyses; Embase: Excerpta Medica database

Eligible articles were selected after multiple steps, as shown in Figure 1. Using the fully developed search strategy, the initial literature search yielded 967 records after removing duplicates. Screening titles and abstracts led to the exclusion of 926 records. Subsequently, 41 articles underwent full-text screening, from which 16 articles were excluded. The primary reasons for exclusion were that the articles were single-arm studies (nine), reviews (four), or used different methods for estimating chronological age (three). Finally, 25 articles were eligible for inclusion in the systematic review, with 21 articles included in the final analysis [2, 3, 26, 27, 31, 33, 34, 40-55].

Study Characteristics

All eligible articles compared the Nolla and Demirjian methods with a total number of participants equal to 15,969. All studies took place between 2010 and 2019. Twenty-four studies reported the gender distribution, up to 7,898 males and 7,871 females. The majority of studies took place in Spain (three), India (seven), and Turkey (four). The conclusions from eligible studies can be summarized as follows: The Demirjian method was favored for older age groups (10-16 years) compared to the Nolla method in the Spanish population. The Nolla method was more accurate for age estimation in three- to 11-year-old western Indian children and showed better accuracy than the Demirjian method in the eastern Turkish and northeastern Turkish populations. The Demirjian method tended to overestimate age, whereas the Nolla method tended to underestimate it. The two methods were valid in some populations, like for children in Nepal and Pakistan. The details of baseline information can be found in Table 1.

**Table 1:** Summary and baseline characteristics of the included studies OPGs: orthopantomogram; NR: not reported

S.No.	Study ID	Study arms	Numbers included	Study setting	Study design	Boys/ Girls	Study period	Inclusion criteria	Conclusion
1	Cortés et al., 2020 [54]	Demirjian	604	Spain	Comparative cross-sectional study	302/302	Between January and December 2017	1. Between January and December 2017; 2. Panoramic radiographs of 604 patients of Spanish ethnicity; 3. Between 4 and 13 years of age; 4. OPG of 7 left mandibular teeth	"In the Spanish population, the use of the Demirjian method for legal and medical purposes is frequent. This study reveals that the Willems method is more appropriate due to its greater precision in estimating dental age."
		Nolla							
		Willems							
2	Deo et al., 2022 [34]	Demirjian	100	India	Retrospective study	51/49	NR	1. Full set of mandibular permanent teeth(erupted/unerupted); 2. Age range between 6-16 years; 3. Subjects who are residents of Pune city; 4. Radiographs for diagnostic use	"The results of this study suggest that Demirjian's method is more suitable than Nolla's method for higher age groups 10-13 years and 14-16 years."
		Nolla							
4	Duruk et al., 2022 [2]	Demirjian	1,587	Turkey	A retrospective study	774/813	Between January 2016 and December 2017	1. Between January 2016 and December 2017; 2. Children aged between 3-17 years; 3. Subjects who are residents of Gujarat city; 4. OPG for the left seven mandibular teeth	"Nolla’s method was more accurate in the CA estimation than Demirjian’s method in the Eastern Turkish population."
		Nolla							
5	Hegde et al., 2017 [41]	Demirjian	1,200	India	A retrospective cross-sectional study	699/501	From January 2012 to September 2015	1. From January 2012 to September 2015; 2. Children aged between 5-16 years; 3. Subjects who are residents of Rajasthan city; 4. OPG for the left seven mandibular teeth	"The Willems and Demirjian methods overestimated age, while the Nolla and Häävikko methods underestimated age."
		Nolla							
		Willems							
		Häävikko							
6	Khanal et al., 2018 [48]	Demirjian	177	Thailand	A cross-sectional study	96/81	From January 2017 to December 2017	1. From January 2017 to December 2017; 2. Children aged between 5-15 years; 3. Subjects who are residents of Jorpati city; 4. OPG for the left seven mandibular teeth	"Both methods showed delayed dental age compared to chronological age. Demirjian’s method was more applicable to assess the dental age in Nepalese children compared to Nolla’s method."
		Nolla							
7	Khoja et al., 2014 [27]	Demirjian	403	Pakistan	A retrospective study	176/227	NR	1. Children aged between 8-17 years; 2. Subjects who are residents of Karachi city; 3. High-quality pre-treatment OPGs of 7 left permanent mandibular teeth	"Overall, Willem’s method was identiﬁed as the most valid method for DA estimation in the Pakistani sample."
		Nolla							
		Willems							
8	Kırzıoglu et al., 2011 [50]	Demirjian	425	Turkey	A retrospective study	212/213	NR	1. Children aged between 7-13 years; 2. Subjects who are residents of Karachi city; 3. OPG of the seven left permanent mandibular teeth except for the third molar	"All of the three methods are not completely suitable for Turkish children and establishment of the population-speciﬁc standards is essential and crucial."
		Nolla							
		Häävikko							
9	Limbu et al., 2021 [51]	Demirjian	280	Nepal	A comparative cross-sectional study	140/140	From December 2020 to September 2021	1. From December 2020 to September 2021; 2. Children aged between 5-14 years; 3. Subjects who are residents of Karachi city; 4. With a full complement of mandibular permanent teeth	"This study revealed Demirjian, Nolla and Willems methods can be a valid measure for age estimation among Nepalese children and precise prediction of chronological age can be made from different dental age estimation techniques."
		Nolla							
		Willems							
10	Lopes et al., 2018 [49]	Demirjian	403	Brazil	A cross-sectional study	168/235	NR	1. Children aged between 7-13 years; 2. Subjects who are residents of Karachi city; 3. Absence of abnormal dental conditions, such as impaction and transposition; 4. OPG of the seven left permanent mandibular teeth	"The Nolla method is suitable for Brazilian children when it comes to age estimate with care to growth spurt beginning (around 11 and 12 years). However, the Dermijian method should not be used, because it over-estimated the age in both sexes."
		Nolla							
11	Maber et al., 2006 [52]	Demirjian	946	Britain	A Retrospective cross-sectional study	491/455	NR	1. Children aged between 3-17 years; 2. OPG of the seven left permanent mandibular teeth	For individual teeth using Haavikko’s method, ﬁrst premolar and second molar were most accurate; and more accurate than the mean value of all developing teeth. The 95% conﬁdence interval of the mean was least for a mean of all developing teeth using Haavikko (age 3–13.99 years), followed by identical values for Demirjian and Willems (sexes combined)."
		Nolla							
		Willems							
		Häävikko							
12	Melo et al., 2017 [31]	Demirjian	2,641	Spain	A retrospective, cross-sectional study	1,319/1,322	Between 2010 and 2014	1. Between 2010 and 2014; 2. Children aged between 3-17 years; 3. Subjects who are residents of Tuusuala city; 4. OPG of the seven left permanent mandibular teeth	"The Nolla and Demirjian methods were found to be accurate in estimating chronological age from dental age in a Spanish population. The error was found to be greater in males than in females and involved an overestimation of age with the Demirjian method and an underestimation of age with the Nolla method. A combination of the Nolla and Demirjian methods for estimating chronological age from dental age affords a predictive capacity of over 99%, and is fast and easy to perform, and inexpensive."
		Nolla							
13	Mohammad et al., 2021 [33]	Demirjian	252	Iraq	A retrospective study	122/130	NR	1. Children aged between 6-15 years; 2. Subjects who are residents of Mosul city; 3. OPG of the seven left permanent mandibular teeth	"The present study concluded that Demirjian’s method and Häävikko`s method are not suitable for DA estimation in Mosul city children aged 6-15 years old. Whereas no significant underestimation of DA with Nolla`s method makes it a more accurate and precise method than the others."
		Nolla							
		Häävikko							
14	Mohammed et al., 2015 [55]	Demirjian	660	India	A cross-sectional study	330/330	NR	1. Children aged between 6-16 years; 2. Subjects who are residents of Andhra Pradesh city; 3. OPG of the seven left permanent mandibular teeth	"Nolla’s method was more accurate in estimating dental age compared to other methods. Moreover, all four methods were found to be reliable in estimating the age of individuals of unknown chronological age in South Indian children."
		Nolla							
		Willems							
		Häävikko							
15	Nur et al., 2012 [42]	Demirjian	673	Turkey	A retrospective study	342/331	NR	1. Children aged between 5-16 years; 2. Subjects who are residents of Andhra Pradesh city; 3. OPG of the seven left permanent mandibular teeth	"Nolla method was found to be a more accurate method for estimating DA in northeastern Turkish population."
		Nolla							
16	Qi Han et al., 2020 [53]	Demirjian	2,000	China	A cross-sectional study	1,000/1,000	Between 2015 and 2018	1. Between 2015 and 2018; 2. Children aged between 5-14 years; 3. Subjects who are residents of Xi'an city; 4. OPG of the seven left permanent mandibular teeth	"Although the Demirjian method is frequently used in Chinese subjects for legal and medical purposes, the Willems and Nolla methods were more reliable than the Demirjian method. Among the three methods, the accuracy in the northern Chinese subjects was highest for the Nolla method. Therefore, it is recommended to evaluate the accuracy of different methods before assessing the age in specific populations."
		Nolla							
		Willems							
17	Rai et al., 2006 [46]	Demirjian	75	India	A retrospective, cross-sectional study	40/35	NR	1. Children aged between 5-14 years; 2. Subjects who are residents of Haryana city; 3. OPG of the seven left permanent mandibular teeth	"The result of our study has shown that Williams's method is more accurate followed by Haavikko, Cameriere, Nolla, and lastly, Demirjian method."
		Nolla							
		Willems							
		Häävikko							
		Cameriere							
19	Sinha et al., 2014 [43]	Demirjian	300	India	Observational study	150/150	NR	1. Children aged between 6-15 years; 2. Subjects who are Indian residents; 3. Absence of any clinical medical history that could affect the development of the permanent teeth; 4. A full complement of mandibular permanent teeth	"The results imply that Demirjian’s method applies to all age groups and for both genders with better accuracy than Nolla’s method, which had a limited utility in younger age group. Thus Demirjian’s method is a better method when compared to Nolla’s method in the Northern Indian population:"
		Nolla							
20	Wen et al., 2022 [53]	Demirjian	535	China	A Retrospective Cross-sectional Study	169/266	Between 2014 and 2019	1. Between 2014 and 2019; 2. Children aged between 6-15 years; 3. Subjects who are residents of Mongolia Autonomous Region; 4. Absence of any clinical medical history that could affect the development of the permanent teeth; 5. OPG of the left seven permanent mandibular teeth	"The newly developed method and dental age conversion scales may be more suitable dental age estimation method for northeastern Chinese children."
		Nolla							
21	Yılmaz et al., 2018 [46]	Demirjian	717	Turkey	Observational study	383/334	From 2014 to 2016	1. From 2014 to 2016; 2. Children aged between 10-15 years; 3. Subjects who are residents of Antalya city; 4. OPG of the left seven permanent mandibular teeth	"There was a positive relationship between dental calcification stages and skeletal maturation stages, in the study population. Dental calcification stages of the second mandibular premolar showed the highest positive correlation with the skeletal maturation stages."
		Nolla							
22	Berkvens et al., 2017 [40]	Demirjian	361	Canada	Observational study	182/179	NR	1. Patients aged less than 30 years; 2. Subjects who are residents of the Ontario province; 3. Absence of any clinical medical history that could affect the development of the permanent teeth; 4. Radiographs demonstrating the presence of at least one-third molar	"All three methods were found to be suitable in the estimation of the chronological age using the third molar. However, the Nolla method proved to be the most applicable at estimating age based on derived polynomial regression R2 values ranging from 0.854 to 0.891."
		Nolla							
		Moorrees							
		MFH							
23	Sybil et al., 2020 [44]	Demirjian	100	India	Observational study	NR	NR	1. Between 12 and 25 years; 2. Subjects who are residents of New Delhi city; 3. Absence of any clinical medical history that could affect the development of the permanent teeth; 4. Presence of the left mandibular third molar whether erupted or unerupted on OPG	"All three methods were a good fit to estimate the chronological age of the patients in our study population. Cameriere method showed higher sensitivity and accuracy in estimating legal age of maturity as compared to the other two methods."
		Nolla							
		Cameriere							
24	Tomás et al., 2014 [3]	Demirjian	821	Spain and Portugal	A cross-sectional study	409/412	NR	1. Between 4 and 34 years; 2. Subjects who are residents of Galicia and North of Portugal; 3. Absence of any clinical medical history that could affect the development of the permanent teeth; 4. Presence of the left mandibular third molar whether erupted or unerupted on OPG	"We can estimate chronological age for early and late childhood, through the Nolla and Demirjian methods, with the former showing greater predictive capacities than the latter. The Demirjian method tends to overestimate age and the Nolla method tends to underestimate it, leading to the importance of forming regression equations adapted to the population studied. Nolla and Demirjian formulas adapted to our sample were created as a function of sex and age group."
		Nolla							
25	Willmann et al., 2023 [45]	Nolla	324	Switzerland	Observational study	156/168	Between January and September 2018	1. Between January and September 2018; 2. Between 4 and 20 years; 3. Subjects who are residents of Strasbourg city; 4. Absence of any clinical medical history that could affect the development of the permanent teeth; 5. OPG of both mandibular sides	"The evaluated methods are unable to provide reliable information to determine if an individual is a minor."
		Demirjian							
		Atlas of London							

Risk of Bias

Scores obtained from the STROBE checklist are not a direct gauge of quality; instead, they should be interpreted as a measure of how rigorously authors reported their findings. Overall, most studies scored between 22 and 32, indicating fair quality. One exception was Sybil et al.'s study, which scored 21. Given that this score falls on the borderline between fair and poor quality, it was decided to include it in the review. The summary scores of the STROBE checklist for individual studies are available in Appendix A.

Results of Syntheses

The discrepancy between the Demirjian and Nolla methods in males is showcased in Table 2.

**Table 2:** Comparison of the effect estimates (pooled for age cohorts) of the Demirjian and Nolla methods in males N: number

Demirjian method					Nolla method					p-value
Age group (years)	N. Studies	N. Population	Effect estimate	95% CI	Age group (years)	N. studies	N. Population	Effect estimate	95% CI	
5-5.99	5	202	0.7	(-0.25, 1.65)	5-5.99	5	202	0.11	(-0.12, 0.34)	0.24
6-6.99	6	257	0.71	(0.33, 1.10)	6-6.99	6	257	0	(-0.18, 0.19)	0.001
7-7.99	9	392	0.52	(0.30, 0.75)	7-7.99	9	392	-0.19	(-0.37, -0.01)	<0.00001
8-8.99	10	458	0.37	(-0.02, 0.76)	8-8.99	10	458	-0.35	(-0.56, -0.13)	0.002
9-9.99	9	439	0.4	(-0.01, 0.80)	9-9.99	9	439	-0.4	(-0.67, -0.12)	0.002
10-10.99	10	484	0.54	(0.26, 0.82)	10-10.99	10	484	-0.22	(-0.52, 0.08)	0.0002
11-11.99	10	409	0.39	(-0.06, 0.84)	11-11.99	10	409	-0.42	(-0.77, -0.07)	0.005
12-12.99	10	524	0.48	(-0.02, 0.97)	12-12.99	10	524	-0.24	(-0.63, 0.14)	0.03
13-13.99	10	455	0.37	(-0.10, 0.83)	13-13.99	10	455	-0.19	(-0.65, 0.27)	0.1
14-14.99	8	337	0.22	(-0.78, 1.22)	14-14.99	8	337	-0.14	(-0.46, 0.18)	0.49
15-15.99	4	72	0.32	(0.01, 0.63)	15-15.99	4	72	0.03	(-0.09, 0.15)	0.08
Overall	16	6395	0.5	(0.28, 0.71)	Overall	16	6395	-0.28	(-0.45, -0.11)	<0.00001

**Figure 2:**
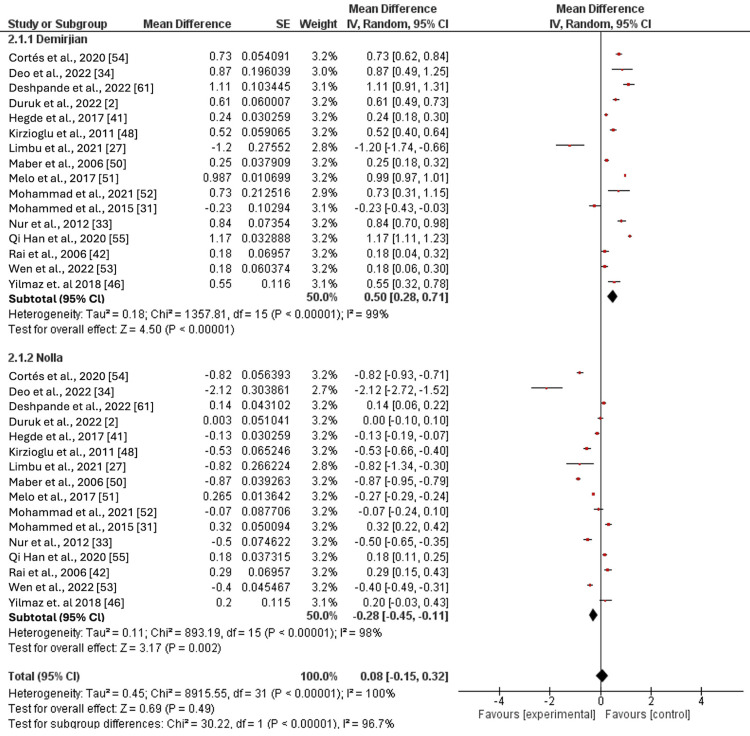
Comparison of the effect estimates (pooled for age cohorts) of the Demirjian and Nolla methods in males Cortés et. al 2020 [54]; Deo et.al 2022 [34]; Duruk et.al 2022 [2]; Hegde et.al 2017 [41]; Kirzioglu et.al 2011 [48]; Limbu et.al 2021 [27]; Maber et. al 2006 [50]; Melo et.al 2017 [51]; Mohammad et.al 2021 [52]; Mohammed et. al 2015 [31]; Nur et. al 2012 [33]; Qi Han et.al 2020 [55]; Rai et.al 2006 [42]; Wen et.al 2022 [53]; Yilmaz et.al 2018 [46]

The Demirjian method overestimated CA by 0.71 years in the age group of six to 6.99 years, which was statistically significant. On the lower end, the overestimation was 0.22 years in the 14-14.99 age group, but this result was not statistically significant. The general trend was that the CA gets overestimated by the Demirjian method by an average of 0.5 years across all age groups combined, and this overestimation was statistically significant compared to the Nolla method. In contrast, the Nolla method tended to underestimate CA in most age groups, with the mean difference ranging from -0.42 years (age group: 11-11.99 years) to -0.14 years (age group: 14-14.99 years). Interestingly, the Nolla method accurately estimated CA in the six to 6.99 age group but slightly overestimated it at the age extremes. On average, the Nolla method underestimated CA by 0.28 years in males, which was statistically significant. Comparing the two methods, a statistically significant difference was observed across most age groups; the Demirjian method overestimated CA, while the Nolla method underestimated it. However, this difference was insignificant in age groups of five to 5.99 years and 13-15.99 years (Figure 2; Appendices B-L)

The difference between the Demirjian and Nolla methods in females is outlined in Table 3.

**Table 3:** : Comparison of the effect estimates (pooled for age cohorts) of the Demirjian and Nolla methods in females N: number

Demirjian method					Nolla method					p-value
Age group (years)	N. Studies	N. Population	Effect estimate	95% CI	Age group (years)	N. studies	N. Population	Effect estimate	95% CI	
5-5.99	5	186	0.69	(-0.19, 1.56)	5-5.99	5	186	-0.1	(-0.26, 0.06)		0.08
6-6.99	6	230	0.69	(0.36, 1.03)	6-6.99	6	230	-0.2	(-0.34, -0.05)	<0.00001
7-7.99	9	359	0.43	(0.21, 0.64)	7-7.99	9	359	-0.5	(-0.63, -0.36)	<0.00001
8-8.99	10	461	0.32	(-0.07, 0.70)	8-8.99	10	461	-0.39	(-0.63, -0.14)	0.003
9-9.99	9	424	0.6	(0.10, 1.09)	9-9.99	9	424	-0.5	(-0.78, -0.22)	0.0002
10-10.99	10	475	0.57	(0.11, 1.03)	10-10.99	10	475	-0.5	(-0.90, -0.10)	0.0006
11-11.99	10	474	0.82	(0.35, 1.28)	11-11.99	10	474	-0.28	(-0.60, 0.04)	0.0001
12-12.99	10	495	0.67	(0.23, 1.10)	12-12.99	10	495	-0.31	(-0.80, 0.19)	0.004
13-13.99	10	471	0.48	(-0.01, 0.98)	13-13.99	10	471	-0.1	(-0.76, 0.55)	0.16
14-14.99	8	346	0.14	(-0.41, 0.69)	14-14.99	8	346	-0.39	(-1.49, 0.71)	0.4
15-15.99	4	91	0.22	(-0.12, 0.57)	15-15.99	4	91	-0.13	(-0.52, 0.27)	0.19
Overall	16	6247	0.54	(0.36, 0.72)	Overall	16	6247	-0.25	(-0.44, -0.06)	<0.00001

**Figure 3:**
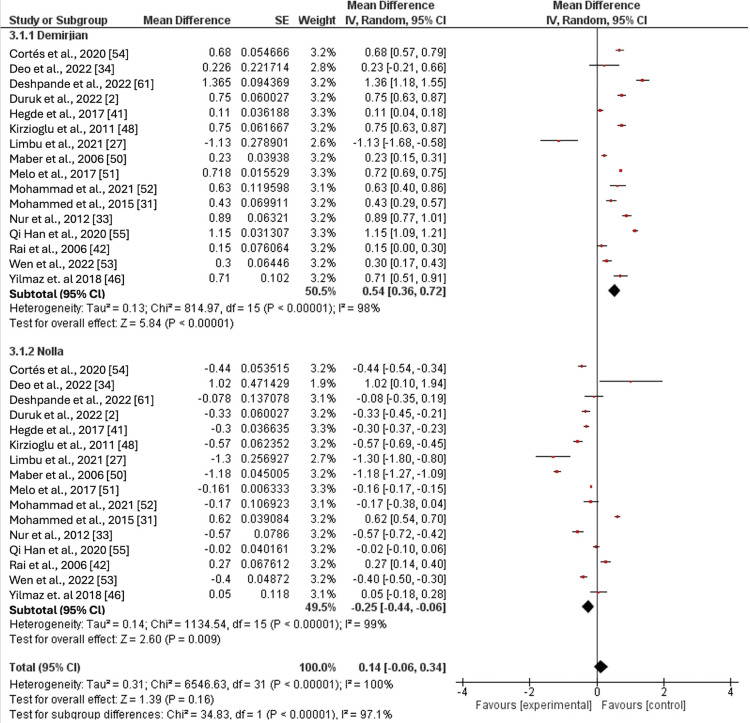
Comparison of the effect estimates (pooled for age cohorts) of the Demirjian and Nolla methods in females Cortés et.al 2020 [54]; Deo et. al 2022 [34]; Duruk et.al 2022 [2]; Hegde et.al 2017 [41]; Kirzioglu et.al 2011 [48]; Limbu et.al 2021 [27]; Maber et.al 2006 [50]; Melo et.al 2017 [51]; Mohammad et.al 2021 [52]; Mohammed et.al 2015 [31]; Nur et.al 2012 [33]; Qi Han et.al 2020 [55]; Rai et.al 2006 [42]; Ven et.al 2022 [53]; Yilmaz et. al 2018 [46]

The CA was overestimated by the Demirjian method by up to 0.82 years in the age group of 11-11.99 years, while the pooled mean difference was 0.14 years in the 14-14.99 age group. Overall, the method had an overestimation of 0.54 years after combining the results from all age groups, which was statistically significant. Conversely, the CA was consistently underestimated by the Nolla method across all age groups, with a mean difference ranging from -0.5 to -0.1 years and a significant pooled mean difference of -0.25 years across all age groups. The Demirjian method showed a statistically significant tendency to overestimate CA in most age groups, whereas the Nolla method consistently underestimated it. However, the difference between both methods was not statistically significant in the age extremes: age groups of five to 5.99 years and 13-15.99 years (Figure 3).

Mean Age Difference Between the Demirjian Method and the Nolla Method in All Groups Combined

**Figure 4:**
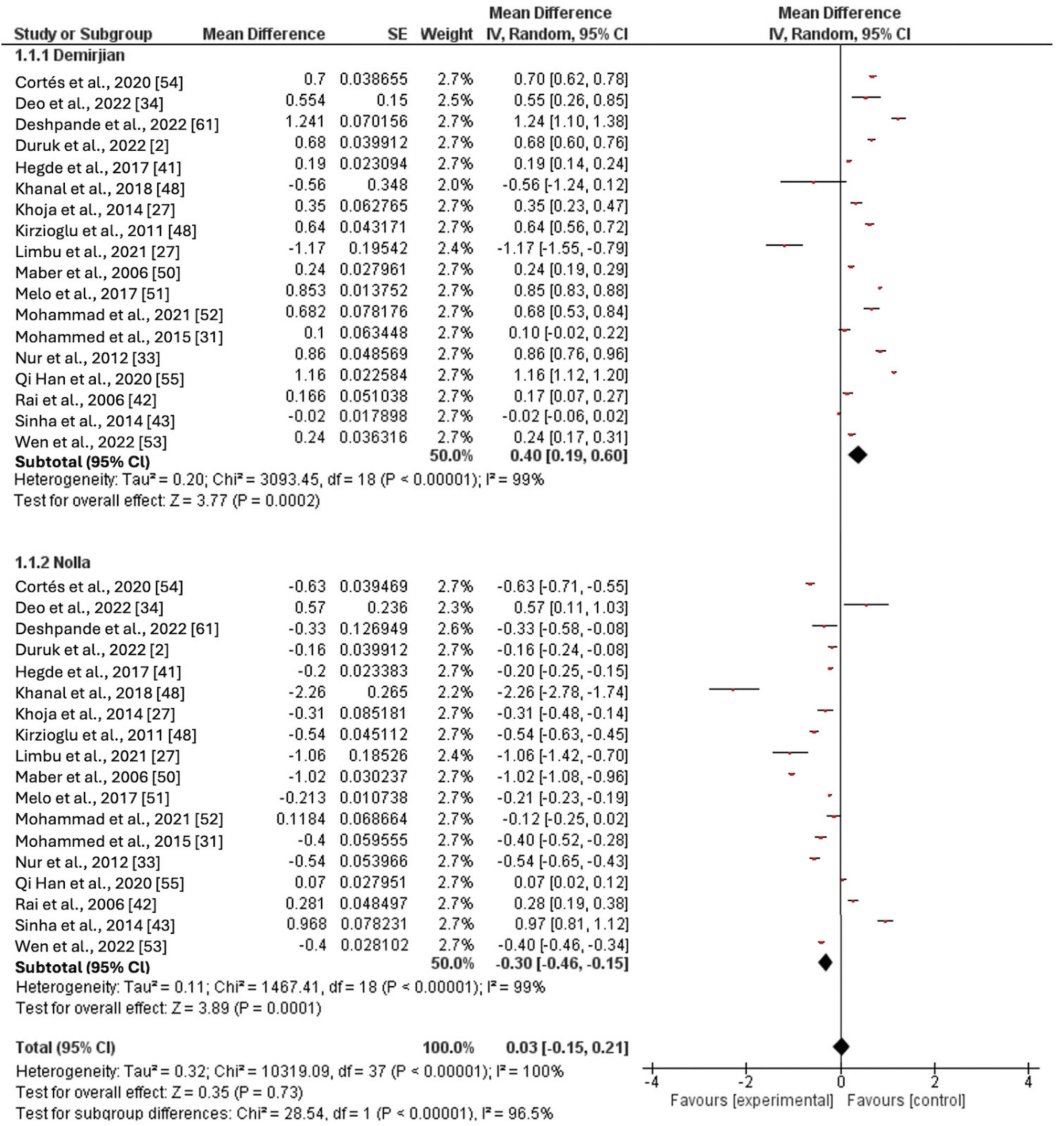
Comparison of the effect estimates (pooled for age cohorts) of the Demirjian and Nolla methods in both genders Cortés et. al 2020 [54]; Deo et.al 2022 [34]; Duruk et. al 2022 [2]; Hegde et.al 2017 [41]; Khanal et.al 2018 [48]; Khoja et. al 2014 [27]; Kirzioglu et.al 2011 [50]; Limbu et.al 2021 [51]; Maber et.al 2006 [52]; Melo et.al 2017 [31]; Mohammad et.al 2021 [33]; Mohammed et.al 2015 [55]; Nur et.al 2012 [42]; Qi Han et.al 2020 [53]; Rai et.al 2006 [46]; Sinha et.al 2014 [43]; Wen et.al 2022 [53]

When combining the male and female results, the Demirjian method consistently overestimated the CA (mean difference (MD): 0.4; 95% CI: 0.19, 0.6; p-value: <0.001). Conversely, using the Nolla method, the CA was underestimated when assimilating the results across all age groups and genders (MD: 0.3; 95% CI: -0.46, -0.15; p-value: <0.001). A statistically significant mean difference of 0.7 years was observed between the two methods when aggregating the data from all age groups (Figure 4).

Discussion

Obtaining an accurate estimate of CA remains a challenging task using available methods from the literature [41, 56, 57]. Dental age estimation provides a better chance of predicting CA than other methods [7, 11]. The Demirjian method is the more widely used method and is well-studied across different populations [3, 12]. Other methods of DA estimation, including the Nolla method, were not the focus of the literature until recently [22]. Here, we compare the performance of the Nolla method in predicting CA to the extensively studied Demirjian method.

Our study compared DA estimates using the Demirjian and Nolla methods to CA, revealing similar patterns in males and females across various age groups. In males, the Demirjian method overestimated CA by an average of 0.5 years, while the Nolla method generally underestimated it by 0.28 years. Similarly, in females, both methods displayed systematic bias; the Demirjian method tended to overestimate age by an average of 0.54 years, and the Nolla method tended to underestimate it by 0.25 years. A significant mean difference of 0.7 years was observed between the two methods. Most included studies reported that the Demirjian method overestimated the CA, except for Limbu et al., whose results showed a statistically significant underestimation [27]. Conversely, most studies showed that the Nolla method underestimated the age. Still, Sinha et al., Han et al., Rai et al., and Deo et al. had statistically significant overestimation of the CA [34, 42, 43, 55].

Contrary to our findings, Franco et al. showed that both methods tend to overestimate CA, but the Nolla method had a lower mean difference. It's important to note that Franco et al. confined the scope of their search to Brazilian studies only [36]. This limited the number of studies in their analysis and may not allow the results to generalize to other populations. Conversely, Willmann et al. found that both the Demirjian and Nolla methods consistently overestimated the age of the subjects. Demirjian's method had a mean absolute error of 1.93 years, significantly higher than Nolla's (1.25 years) [45]. Nevertheless, both studies demonstrated that the Nolla method was more accurate, aligning with our results.

Gender notably affected our estimates produced by both methods, each exhibiting a distinct pattern. The Demirjian method had three clusters, each with a unique trend. The first cluster encompasses ages five to 8.99 years, and the method tends to produce more minor errors in females than males. Ages in the second cluster range from nine to 13.99 years, where males have smaller errors in estimates than females. A third cluster extends between the ages of 14-15.99 years, where the method offers better estimates for females. A similar pattern showed up in Esan et al., where the Demirjian method produced worse estimates in males initially; on the other hand, the results became comparable after the age of seven years. The Nolla method had a general trend of more significant errors in females in all except three age groups [12].

Our results demonstrate that the Nolla method tends to produce smaller errors across most age groups and in both genders; however, exceptions exist for this pattern. In females, the Nolla method performed worse than the Demirjian method in three age groups (seven to 7.99, eight to 8.99, and 14-14.99); this pattern was noticed in males only in the age group 11-11.99. Two previous meta-analyses had findings that match ours. Bittencourt et al. demonstrated that the Nolla method exhibited a stronger correlation with CA than the Demirjian method. Additionally, the Nolla method showed lower heterogeneity in males and comparable heterogeneity in females relative to the Demirjian method [58]. A Croatian study by Berkvens et al. showed that in males, the Demirjian method tends to overestimate age on average scores from all four third molars [40].

In contrast, the Nolla method provides more accurate estimations by considering all four third molars. Berkvens et al. also noted that both methods were comparable in females [40]. Nevertheless, according to Sybil et al., both males and females showed a higher coefficient of determination with the Demirjian method. This suggests that the Demirjian method was more effective than the Nolla method for estimating CA in the North Indian population [44].

The accuracy of dental age estimation methods, such as the Demirjian and Nolla methods, can vary significantly across different ethnic groups due to genetic, environmental, and nutritional factors influencing dental development. This variability can lead to discrepancies in age estimation if the methods are applied universally without considering ethnic-specific differences. Practitioners should use ethnic-specific standards and reference data when available to enhance accuracy. In the absence of such data, caution should be exercised, and the broader context of the case should be considered when interpreting results. Further research is needed to develop and validate age estimation methods across diverse ethnic populations, which will help establish more accurate and reliable standards for dental age estimation.

Noteworthily, the Demirjian method performs much better in older age groups than younger age groups. On the other hand, the Nolla method tends to perform better on either extreme of the spectrum. In 2014, Tomas et al. conducted a study comparing the predictive value of each method, taking into account the effect of gender and the variations across different age groups. The Nolla method generally outperformed the Demirjian method for younger ages, with a 24.2% better forecasting rate for children aged between four and 10. However, as children aged, both methods offered comparable results. For males up to 15 years, the Nolla method was superior, but for older teenagers, the Demirjian method was more effective. Similarly, Nolla was more accurate in females up to 10 years, while Demirjian showed better accuracy from late childhood to 18 years of age [3].

The application of dental age estimation methods can vary significantly depending on the forensic context. For instance, in immigration cases where an individual's age is disputed, an age underestimate might be more appropriate as it could favor the individual. The Nolla method, which tends to underestimate age, could be particularly useful in these scenarios, ensuring that individuals are not unfairly treated or denied rights based on an overestimated age. Conversely, in cases involving the identification of found human remains, a broader age estimate may be more helpful in locating missing persons' records. The Demirjian method, which tends to overestimate age, can provide a wider age range that might match a broader set of missing persons' profiles. This broader result can aid in narrowing down potential matches and facilitate the identification process. By considering the specific context of each forensic case, practitioners can tailor their approach to age estimation, enhancing the accuracy and fairness of the process. This contextual application underscores the importance of selecting the appropriate method based on the forensic scenario, thereby improving dental age estimation techniques' practical relevance and impact.

Heterogeneity was very high across all models since different countries have intra- and inter-population variations because of ethnicity, genetics, gender, and other environmental factors affecting the normal development of teeth. Dental age estimation comes with a set of challenges impacting its accuracy. Many studies highlighted the variability in tooth development influenced by genetic, environmental, and nutritional factors, leading to discrepancies between estimated and CA, especially when dental development deviates from typical patterns [15, 16, 19]. Additionally, ethnicity is pivotal in age estimation precision because of inter-ethnic differences in dental maturity timings and rates [17]. Ethnic-specific standards have been recommended to enhance the accuracy of DA estimation methods [59, 60, 61].

To the best of our knowledge, this work is the first to compare the performance of the Demirjian and Nolla methods from all available literature. We addressed many issues raised by previous literature, such as the effect of gender and the considerable variations across different age groups. However, we could not account for the effect of ethnicity due to the limited number of studies within each category.

## Conclusions

In conclusion, while the Demirjian and Nolla methods each have their unique advantages, using them together can provide a more robust and reliable age estimation. Forensic practitioners can determine the best approach by considering population-specific accuracy, age group and gender, and the context of the case. The combined use of both techniques offers cross-verification, comprehensive assessment, bias mitigation, and enhanced reliability.

## Figures and Tables

**Figure 5:**
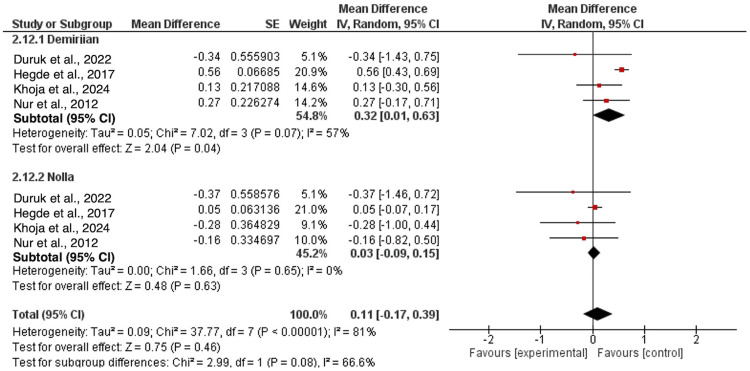
Comparison of the effect estimates (age cohort of 15 to 15.99 years) of the Demirjian and Nolla methods in males

**Figure 6:**
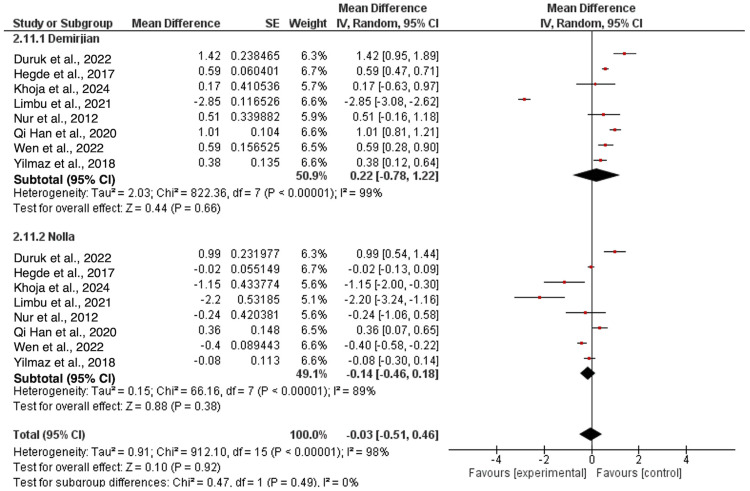
Comparison of the effect estimates (age cohort of 14 to 14.99 years) of the Demirjian and Nolla methods in males

**Figure 7:**
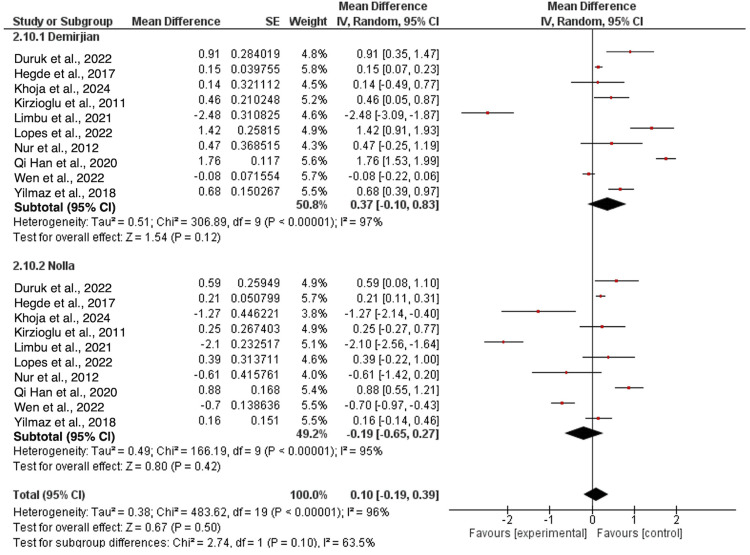
Comparison of the effect estimates (age cohort of 13 to 13.99 years) of the Demirjian and Nolla methods in males

**Figure 8:**
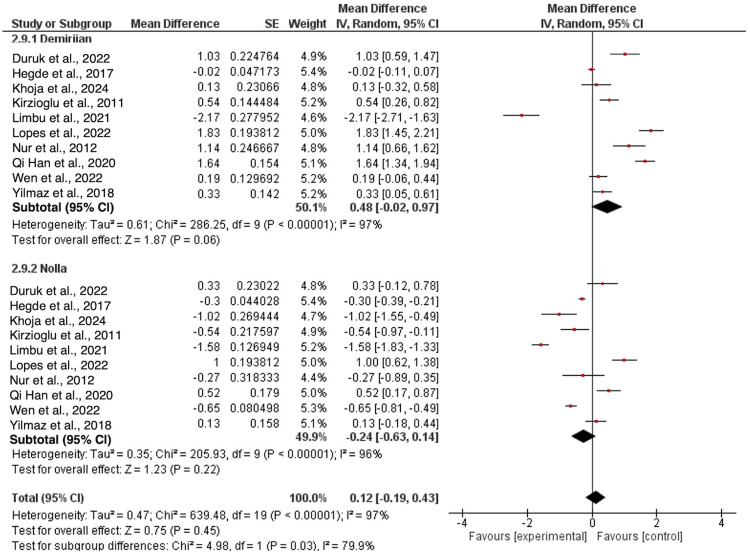
Comparison of the effect estimates (age cohort of 12 to 12.99 years) of the Demirjian and Nolla methods in males

**Figure 9:**
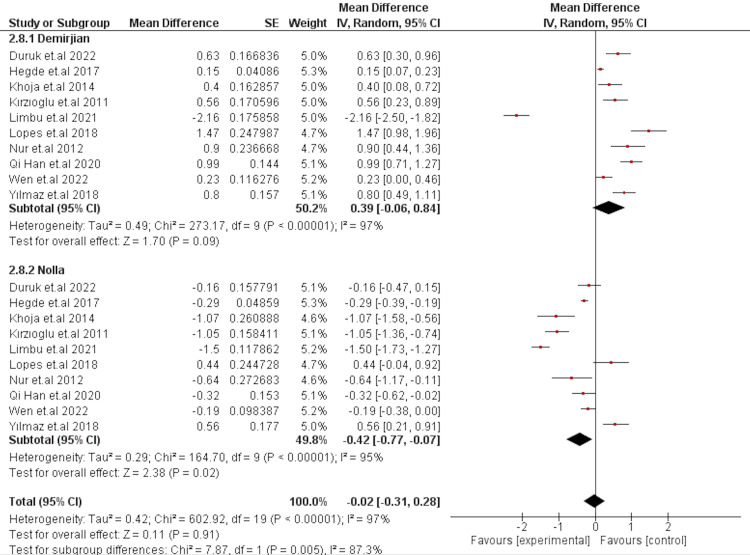
Comparison of the effect estimates (age cohort of 11 to 11.99 years) of the Demirjian and Nolla methods in males

**Figure 10:**
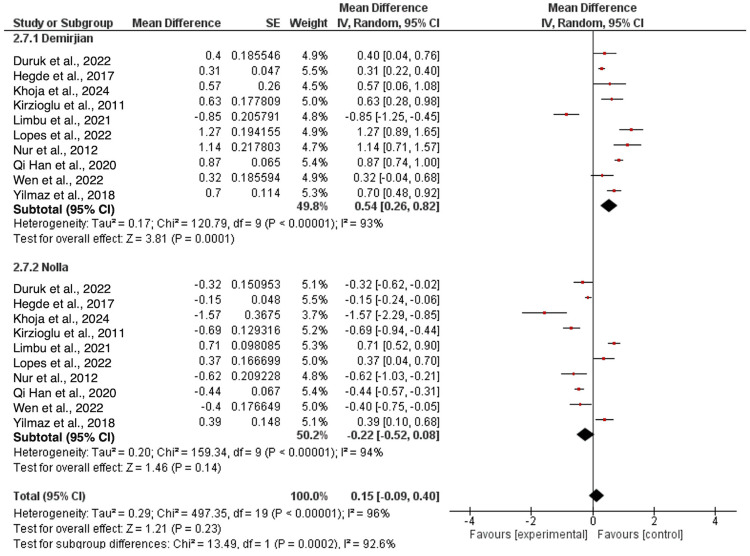
Comparison of the effect estimates (age cohort of 10 to 10.99 years) of the Demirjian and Nolla methods in males

**Figure 11:**
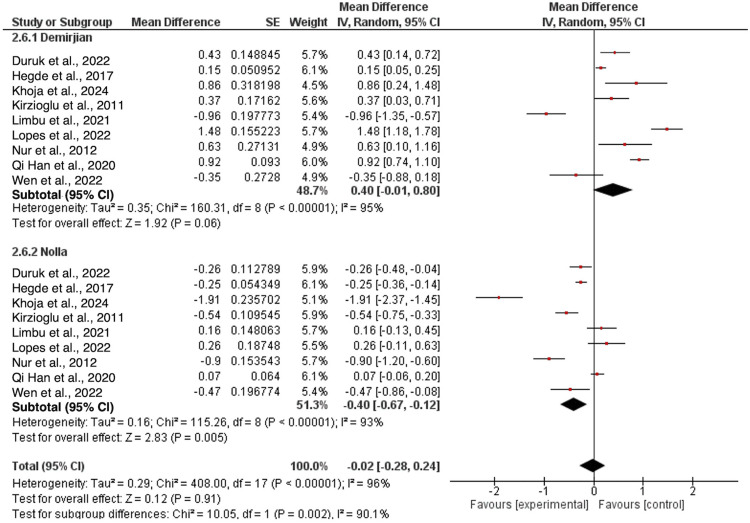
Comparison of the effect estimates (age cohort of 9 to 9.99 years) of the Demirjian and Nolla methods in males

**Figure 12:**
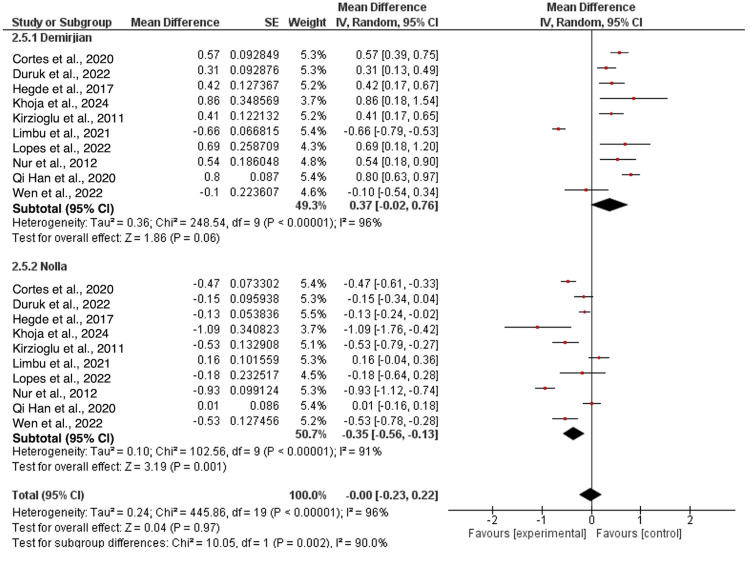
Comparison of the effect estimates (age cohort of 8 to 8.99 years) of the Demirjian and Nolla methods in males

**Figure 13:**
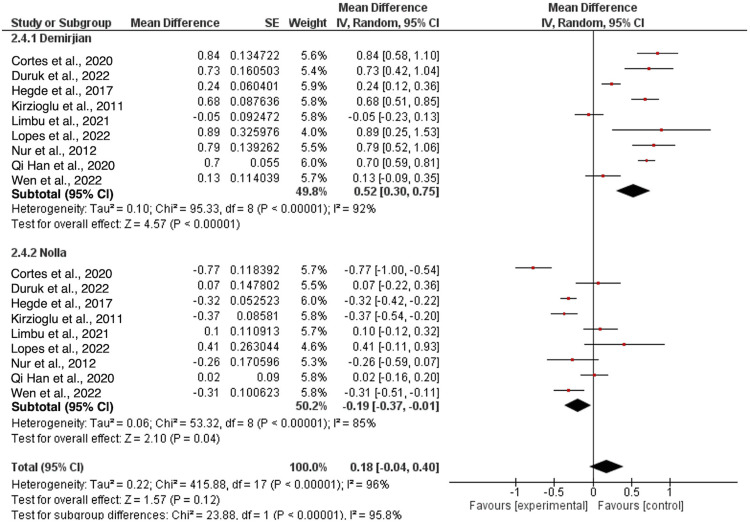
Comparison of the effect estimates (age cohort of 7 to 7.99 years) of the Demirjian and Nolla methods in males

**Figure 14:**
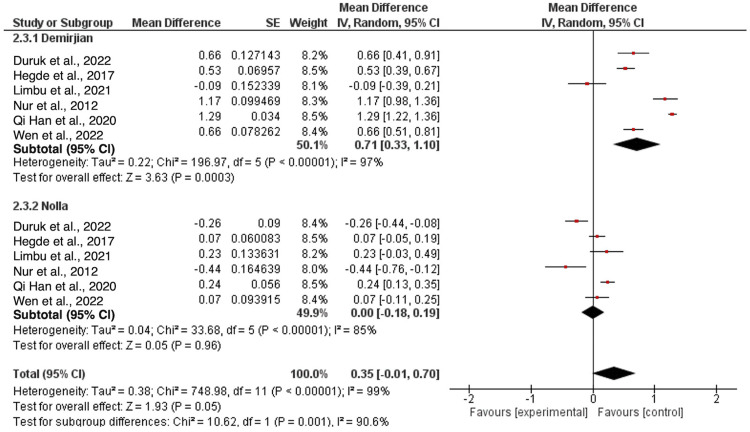
Comparison of the effect estimates (age cohort of 6 to 6.99 years) of the Demirjian and Nolla methods in males

**Figure 15:**
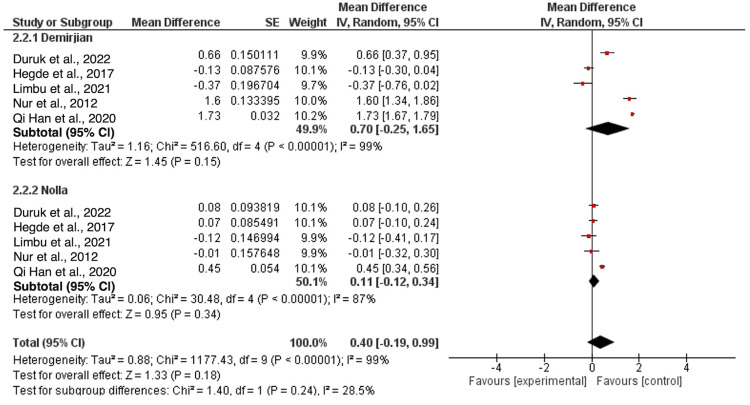
Comparison of the effect estimates (age cohort of 5 to 5.99 years) of the Demirjian and Nolla methods in males

## Appendices

Appendix A 

**Table 4:** STROBE 40-item checklist scores for included studies STROBE: Strengthening the Reporting of Observational Studies

Study ID	Study design	Score
Cortés et al., 2020	Comparative cross-sectional study	32
Deo et al., 2022	Retrospective	24
Deshpande et al., 2022	Cross-sectional study	27
Duruk et al., 2022	Retrospective	29
Hegde et al., 2017	Cross-sectional study	27
Khanal et al., 2018	Cross-sectional study	22
Khoja et al., 2014	Retrospective	29
Kırzıoglu et al., 2011	Retrospective	26
Limbu et al., 2021	Comparative cross-sectional	31
Lopes et al., 2018	Cross-sectional study	27
Maber et al., 2006	Cross-sectional study	22
Melo et al., 2017	Cross-sectional study	29
Mohammad et al., 2021	Observational	29
Mohammed et al., 2015	Cross-sectional study	25
Nur et al., 2012	Retrospective	30
Qi Han et al., 2020	Cross-sectional study	29
Rai et al., 2006	Observational	22
Sabin et al., 2018	Retrospective	20
Sinha et al., 2014	Observational	26
Wen et al., 2022	Cross-sectional study	29
Yılmaz et al., 2018	Observational	26
Berkvens et al., 2017	Retrospective	25
Sybil et al., 2020	Pilot study	21
Tomás et al., 2014	Cross-sectional study	25
Willmann et al., 2023	Observational	23

Appendix B 

Appendix C 

Appendix D 

Appendix E 

Appendix F 

Appendix G 

Appendix H 

Appendix I 

Appendix J 

Appendix K 

Appendix L 
